# Correlations between B-mode ultrasound image texture features and tissue temperatures in hyperthermia

**DOI:** 10.1371/journal.pone.0266446

**Published:** 2022-10-06

**Authors:** Xuelin Wang, Lei Sheng

**Affiliations:** 1 School of Engineering Medicine, Beihang University, Beijing, China; 2 Key Laboratory of Cryogenics, Technical Institute of Physics and Chemistry, Chinese Academy of Sciences, Beijing, China; University Tunku Abdul Rahman, MALAYSIA

## Abstract

**Purpose:**

The noninvasive monitoring of mild hyperthermia or thermal ablation is important to guarantee therapeutic safety and efficacy. The potential of ultrasound B-mode image texture features in monitoring temperature or coagulation zones studied in this article.

**Materials and methods:**

The experiments carried out on eighteen *in vitro* porcine liver samples heated from 20°C to 60°C in the water bath. The ultrasound radiofrequency signal at different temperature collected to reconstruct B-mode ultrasound images. The texture features based on gray level histogram (GLH), gray level co-occurrence matrix (GLCM), and gray level-gradient co-occurrence matrix (GGCM) extracted, respectively. Accordingly, we analyze the correlations between these texture features and temperature based on the experiment results.

**Results:**

The results showed that five texture feature parameters closely related to temperature, including mean gray scale of GLH, homogeneity of GLCM, hybrid entropy, inverse difference moment, and correlation of GGCM. Some of these feature parameters have correlation coefficients larger than 0.9 within the temperature range of 20°C to 60°C.

**Conclusions:**

The above-mentioned five feature parameters expected to apply for noninvasive monitoring of MH or TA.

## 1. Introduction

Hyperthermia has become a new technology for tumor treatment in recent years, which divided into mild hyperthermia (MH) and thermal ablation (TA). The MH heats the tumor to 42–45°C and maintains for certain time to inhibit tumor growth, which commonly used as an adjuvant to radiotherapy or chemotherapy [1]. The TA generates a locally high temperature (> 60°C) in a short time to make coagulative necrosis of tumor tissues for in-situ inactivation or partial eradication [2]. However, unlike the conventional open surgery in which the tumor is visible during treatment. MH or TA requires imaging equipment to monitor the effect of treatment and inform the physician of whether the tumor treated or not. Compared with MRI, the Ultraonud (US) has the advantages of low cost, real-time data acquisition and processing capability, and good human-body penetrability. Therefore, ultrasonic techniques have been a hot topic in the research field of noninvasive thermal therapy monitoring [3]. Ultrasonic monitoring of hyperthermia includes two aspects, i.e., temperature, and coagulation zones or thermal lesions. The MH monitoring deals with temperature estimation only, while the TA monitoring requires both temperature estimation and thermal lesions detection. Ultrasonic monitoring techniques employ acoustic parameters mainly based ultrasound elastography [4] and backscattering features [5–7], These acoustic parameters may be closely related with the temperature range in conventional hyperthermia (42–45°C). However, at high temperature, especially when the tissue coagulated, the echo shift and backscattered energy have little variation with the change of temperature, and consequently it is hard to monitor the temperature change of TA based on the existing ultrasonic temperature monitoring techniques.

Fortunately, B-mode ultrasound images can be acquired to monitor treatments during the entire procedure of hyperthermia [8], which are always used for the guidance of ablation therapies [9]. Wang et al. [10] using texture feature of B-mode ultrasound images to find the differences between fresh and frozen-thawed ex-vivo porcine liver tissue shows that image texture can be used for tissue characterization. Li et al [11] demonstrated that the mean gradient and mean gray scale of B-mode ultrasound images increase with the increase of tissue temperature during microwave ablation. They also proposed the possible use of B-mode ultrasound images in noninvasive monitoring of temperature changes in hyperthermia. There is a strong correlation between texture parameters of ultrasound images and tissue temperature. To deal with the problem that most of the current ultrasonic hyperthermia monitoring methods are not applicable for TA, this paper systematically studies the majority of texture parameters to explore the most significant ones that used for noninvasive monitoring of both MH and TA. To accomplish this goal and eliminate the impacts of ROI selection on texture parameters, the temperature-controlled water bath heating experiments are conducted on account of the reason that the water bath heating can achieve homogenous temperature field and coagulation.

## 2. Materials and methods

### 2.1. Experimental apparatus and data acquisition

**Fig 1:**
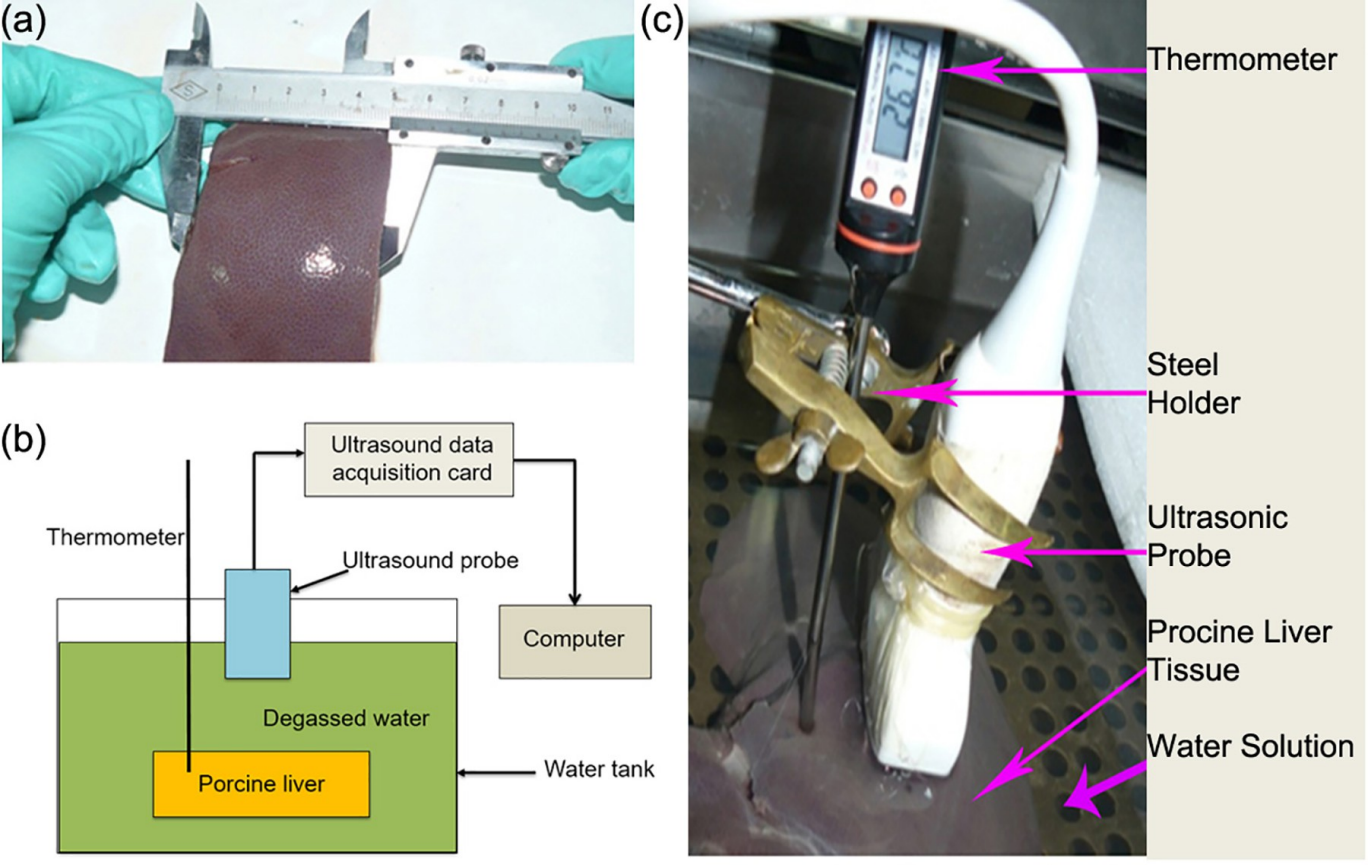
Experimental acquisition of ultrasonic images for porcine liver tissues. (a) Measure the porcine liver tissue to control the size of each sample. (b) The schematic diagram of the experimental system. (c) The set-up of the experimental system, when the temperature reading of the electronic thermometer was the same as that of the water tank thermometer, the temperature inside the porcine liver became uniform, and then the ultrasound echo signal collected and stored in the computer for further data analysis.

As the porcine livers have similar compositions as human’s, they were chosen as the experimental materials. Fresh porcine livers bought from a slaughterhouse; each of them cut into two cubic samples (6×5×4 cm), as shown in Fig 1A. The schematic diagram of the experimental system shown in Fig 1B. Eighteen *in vitro* porcine liver samples applied, and each sample thoroughly immersed in the solution to improve uniform heating. The ultrasound probe stabilized by a holder and placed orthogonal to the porcine liver sample. Besides, the thermometer inserted into the samples 1 cm deep. The system setup shown in Fig 1C. The ultrasound device was TH-600 equipped with an original radiofrequency (RF) signal output port. The temperature range of the thermometer (TP3001, the length of probe was 150 mm, and the diameter of probe was 21 mm) was from 0°C to 200°C, with the resolution of 0.1°C. The thermostatic water tank was HH-W21-600C from Beijing Changfeng Instrument Company, China, and the temperature fluctuation and uniformity of the tank was within ±0.5°C.

**Fig 2:**
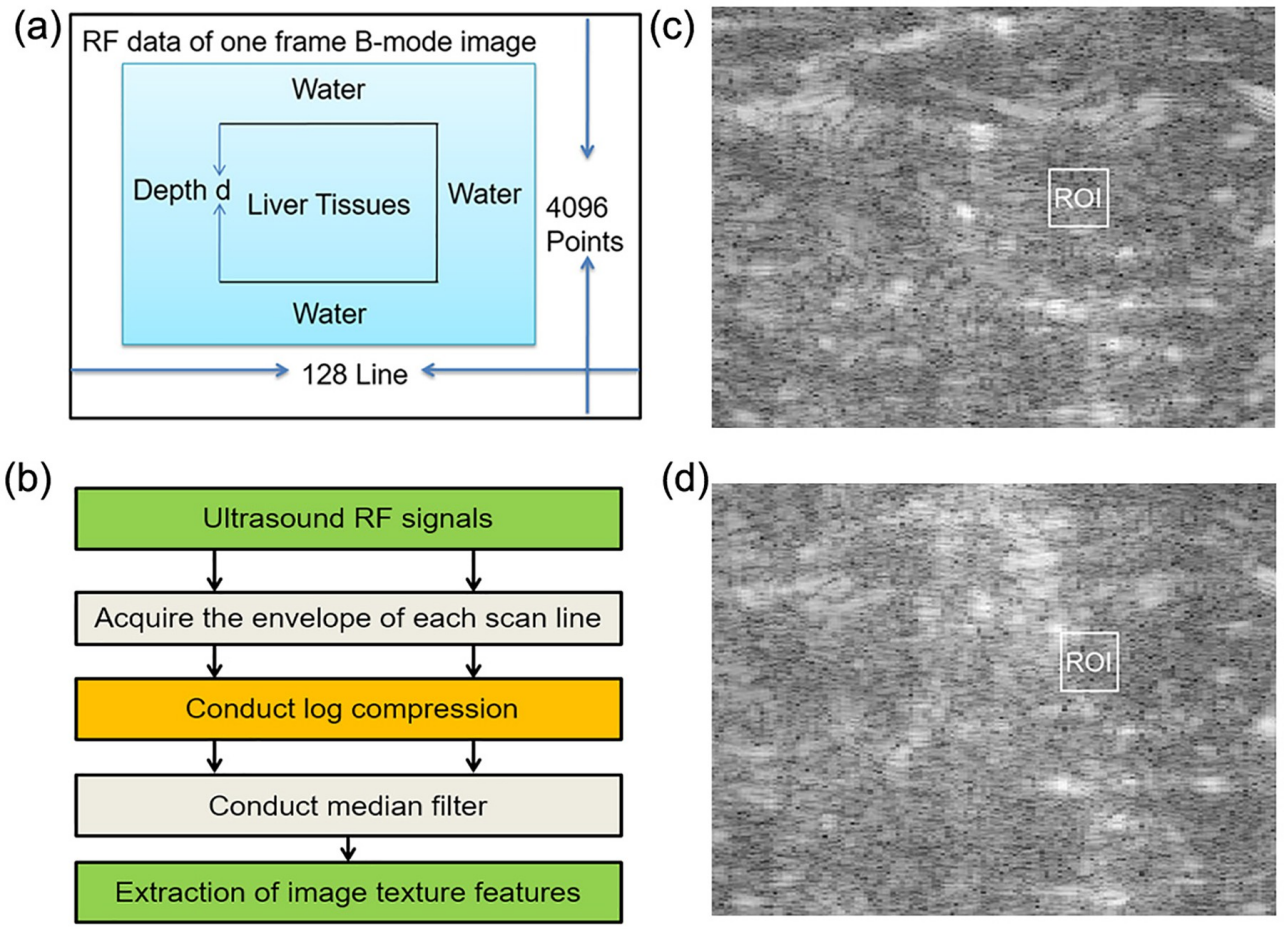
Reconstruction of B-mode images. (a) The ultrasound RF data of one frame B-mode image. (b) Reconstruction B-mode ultrasound image of liver tissue at 20°C during water-bath experiment which represented normal structure, and ROI was selected manually from the images for each sample. (c) Steps for image reconstruction from ultrasound RF signals. (d) Reconstruction B-mode ultrasound image of liver tissue at 60°C during water-bath experiment which represented coagulated structure, and ROI was selected manually from the images for each sample.

The modified diagnostic ultrasound system consisting of one 128-element linear array imaging probe at the central frequency of 3.5 MHz (TH-600, Teknova Medical Systems Limited, China) was utilized to capture RF signals backscattered by the samples and digitize RF signals at a sampling rate of 14MHz with the precision of 16 bits, as shown in Fig 2A. To obtain the ultrasound image of porcine liver at a certain temperature, the water bath experiment conducted. When the temperature reading of the electronic thermometer was the same as that of the water tank thermometer, it assumed that the temperature inside the porcine liver became uniform. To ensure the uniformity of temperature distribution, we used 5 minutes for the temperature to rise by one degree Celsius in pre-warming, therefore, from 20°C to 60°C, it took about 200 minutes for one sample in the whole experiment process. The ultrasound echo signal collected and stored in computer for further data analysis.

### 2.2. Fundamental physical mechanism

The physical properties of the tissue used in diagnostic ultrasound originates from scattering processes. Tissue identification and clinical diagnosis performed by the observation of scattered ultrasound [12]. The B-mode contrast observed from tissue is caused by physical phenomenon called “ultrasound scattering”, and the scattering properties of tissues can be defined quantitatively by using two statistical parameters, i.e., velocity fluctuation coefficient and correlation length [13]. Ultrasound speed is correlate with tissue temperature. The ultrasound transducers receive backscattered signals whose envelope results into B-mode images. B-mode ultrasound image textures are form based on the differences among ultrasound absorption, attenuation, and reflection in various tissue fibers. Therefore, the images of different tissues can show different special texture characteristics. Thermal effects caused by MH or TA may result in hyperemia, vesicular, gasification, coagulation, carbonization, etc., as ablation continues. These effects further lead to the changes in tissue structures and properties. In short, image textures can change during MH or TA. In the case that the B-mode image has temperature dependency, the image textures correlated with temperature.

### 2.3. Reconstruction of B-mode images

Medical ultrasound B-mode imaging is a popular detection method, which can clearly reflect the cross-sectional anatomy of a tissue with important clinical value. As shown in Fig 2B, the steps for the reconstruction of the ultrasound B-mode images from original RF signal are as follows:

(1) Acquire the envelope of RF signal of each scan line. To reduce the data distortion due to mathematical transformation, the most original method utilized to reconstruct the ultrasound image, i.e. using the maximum value of certain length of data to detect the envelope instead of Hilbert and other mature algorithms. To improve the resolution of reconstructed ultrasound image, the method of the maximization of 4 points used to obtain more image texture information for analysis. The reconstructed image consists of 128 scan lines, each of which has 512 sample points. B-mode images reconstructed from ultrasound echo envelope and the envelope of each scan line calculated by the following formula:

b(i)=max(echo(i−1)×4+j),0≤j≤4,0≤j≤512 (1)

Where *b*(*i*) denotes the envelope, and *echo*(*i*) represents the RF echo. To obtain finer image data, the experimental depth was set as half of the detection depth, the sampling depth of the ultrasound device was 19.2 cm and the depth of the experimental setup was about 10 cm. The first 2,048 points of the 4,096 points used to obtain more image texture information for analysis.

(2) Conduct log compression and threshold detection. The depth of ultrasound RF data is 16-bit, the dynamic range of the envelope signal is therefore too large to be displayed. Logarithmic compression can reduce the dynamic range of the envelope signal and retain the original signal information to a certain extent. The 16-bit data was thus log-compressed to 8-bit data for display. After logarithmic compression, as the envelope value has to be limited to the range from 0–255, threshold detection adopted for display, and threshold detection calculated by using the following formula:

$$b(i)=\{\begin{array}{ll} b(i) & b(i)\geq \mathrm{Thresh}\\ 1 & b(i)<\mathrm{Thresh} \end{array}$$ (2)

where *b*(*i*) denotes the envelope, *Thresh* stands for the selected threshold, and the logarithmic compressed values were mapped to the range of 0–255 according Formula 3 where *b(max)* refers to the maximum amplitude of the data set.


$$b(i)=\mathrm{Min}\{{\left[b(i)/b(\max)\right]}^{*}256,255\}$$ (3)

As the dynamic range of the data was too large, some low-amplitude points were in black after display, which stand for the speckle noise.

(3) Conduct median filter. In the process of ultrasound image reconstruction, due to the great difference of acoustic impedance at the tissue boundary, the envelope shape showed obvious peaks, and its amplitude was generally larger. Some low-amplitude peaks usually form speckle noise on B-mode ultrasound images. Although the removal of low-threshold signals can suppress some speckle noise, it is possible to filter out the useful information. Because these noises caused by some low-amplitude points and most of them were not from porcine liver tissue, filtering out these noises had minor effect on the results. To make the ultrasound image clearer, the median filter used to reduce the noises and retain original RF information. The reconstructed B-mode images of normal and coagulated structure shown in Fig 2C and 2D respectively.

### 2.4. Extraction of image texture features

Texture is one of the important characteristics used in identifying and characterizing objects or regions of interest in an image. The commonly-used texture analysis approaches are based on the probability distribution of gray levels and the texture pattern properties. The features measured from the first-order and second-order statistics. In this paper, temperature distribution of the samples was uniform due to utilization of the water bath system, and ROIs selected randomly from the images for each sample as long as the ROI is within the tissue. The size and depth of ROIs have no impact on the experimental results due to the usage of linear array imaging probe. ROIs selected manually. The result would suffer subjective interpretation and possibly human errors. Regions of 16×16 pixels selected as ROIs on each reconstructed B-mode image, as shown in Fig 2C and 2D, respectively. In this study, 3 gray level histogram (GLH) based features, 16 texture parameters of gray level co-occurrence matrix (GLCM) at 4 directions, and 12 texture parameters of gray level-gradient co-occurrence matrix (GGCM) were used for the analysis of temperature correlation.

#### 2.4.1. Textural features based on the GLH

The GLH method applied to extracts the first order statistical texture features describing the probability of occurrence of grey scale in an image, and reflecting the periodicity and density of image texture structures. The three parameters that were analyzed include: the mean of the grey scale (MGS), variance (VAR) and entropy (ENT); MGS describes the average grey level of a region so that it can provide a rough idea of intensity; VAR is commonly known as ‘second moment’, which measures grey-level contrast, and is useful for the description of relative smoothness. While ENT describes the randomness of an image, which calculated by using the following formula:

$$ENT=\underset{i}{\sum }P_{i}{\mathrm{Log}}_{2}P_{i}$$ (4)

Where P_i_ refers to the probability that the difference between two adjacent pixels is equal to i, and Log_2_ is the base 2 logarithm.

#### 2.4.2. Texture features based on the GLCM

GLCM extracts the second order statistics from an image, which is widely used for texture classification [14]. Haralick [15] defined GLCM as a matrix of frequencies at which two pixels, separated by a certain vector, occur in the image. The distribution in the matrix depends on the relationship of angular (θ) and the distance (d) between pixels. The variation of the vector used allows the capturing of different texture characteristics. Once the GLCM created, various features computed from it, which classified into four groups, i.e., visual texture characteristics, statistics, and information theory and information measures of correlation [16]. Table 1 lists the four commonly used features. Before calculating features, each GLCM matrix normalized through dividing each element by the total number of elements. Therefore, each element (r,c) was the joint probability occurrence of pixel pairs which have a defined spatial relationship with grey level values r and c in the image [17].

**Table 1:** Textural features calculated from the normalized co-occurrence matrix.

Feature	Formula
Contrast	$\sum_{i=0}^{L-1}\sum_{j=0}^{L-1}{\left(i-j\right)}^{2}P(i,j)$
Correlation	$\frac{\sum_{i=0}^{L-1}\sum_{j=0}^{L-1}(i-\mu_{x})(j-\mu_{y})P(i,j)}{\sigma_{x}\sigma_{y}}$
Energy	$\sum_{i=0}^{L-1}\sum_{j=0}^{L-1}P^{2}(i,j)$
Homogeneity	$\sum_{i=0}^{L-1}\sum_{j=0}^{L-1}\frac{P(i,j)}{1+{\left(i-j\right)}^{2}}$

Where *μ*_*x*_, *μ*_*y*_, *∂*_*x*_ and *∂*_y_ are the means and standard deviations of *P*_x_ and *P*_y_ respectively, with *P*_x_ is the sum of each row and *P*_y_ is the sum of each column in the co-occurrence matrix.

The normalized probability density p_∂_(*i*,*j*) of the co-occurrence matrices can be defined as:

$$p_{\partial }(i,j)=\frac{\#\{|\left(x,y),(x+d,y+d\right)|\in S|f(x,y)=i,f(x+d,y+d)=j\}}{\#S}$$ (5)

where x,y = 0,1,… ..N-1 refer to the coordinates of the pixel, i,j = 0,1,… .L-1 represent the grey levels, S stands for the set of pixel pairs which have certain relationship in the image, #S denotes the number of elements in S, p_∂_(*i*,*j*) is the probability density of the first pixel to have intensity value i and the second j, which is separated by distance d = (dx, dy), and dx and dy refer to the infinitely small changes in x and y directions respectively [17].

Seen from Eq 5, a GLCM is a matrix where the number of rows or columns is equal to that of grey levels L in the image. In this study, the B-mode ultrasonic images were 8-bit grey level images (256 grey levels), so L was set to 16 for texture differentiation, and d was set to 1 for a better texture analysis. It hypothesized that a single GLCM is not enough to describe the textural features. For example, a single horizontal offset might not be sensitive to texture with a vertical orientation. Therefore, GLCMs were created along four directions (α = 0°, 45°, 90°, 135°) of the ROI in this experiment. After that, each GLCM normalized and four parameters (Contrast, Correlation, Energy and Homogeneity) were calculated.

#### 2.4.3. Texture features based on the GGCM

The GGCM is the combination of the gray level histogram and the edge gradient histogram, which reflects the correlation between gray levels and gradients. Based on normalized GGCM, a series of quadratic statistical features can be calculated, and the feature parameters based on the GGCM were selected, including small gradient preponderance, large gradient preponderance, gray level nonuniformity, gradient nonuniformity, energy, mean gradient, gradient mean square deviation, correlation, gradient entropy, hybrid entropy, and inverse difference moment [18].

## 3. Results

In this study, the original ultrasound RF signals from 20°C to 60°C at the increment of 1°C collected to diminish device-dependent variances. 18 in vitro porcine liver samples were applied, and the texture features of the reconstruction B-mode images under various temperatures were extracted and analyzed for each sample. Besides ROIs selected at each experiment and 31 image texture parameters obtained at this temperature, for example, at 20°C. For each case of experiment, the temperature dependence of each parameter calculated separately based on individual differences. Correlation coefficient, generated by the Pearson product-moment correlation used to measure the linear relationship between each parameter and temperature. Statistical correlation coefficients were expressed as Average-Value ± Std for 18 experiments, and Std represents the standard deviation of statistical correlation coefficients. All the coefficient values in the table are significant (p < 0.05), besides, the slopes of Average-Value are around one, which considered as a good fit.

### 3.1. Correlations between GLH based texture features and temperature

**Fig 3:**
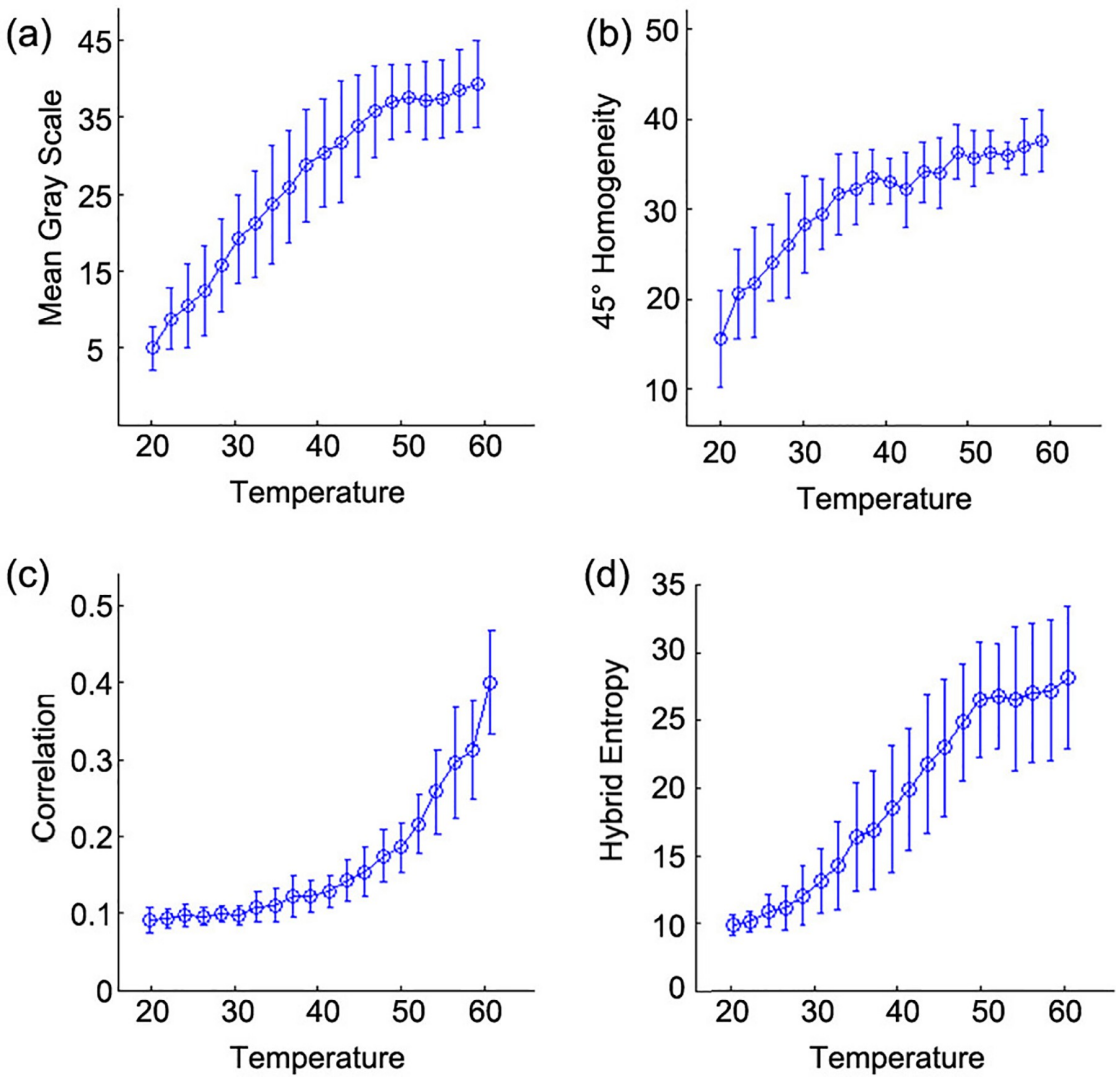
Changes of the B-mode ultrasound image texture features with temperature. (a) Average Value±Std of the mean gray scale changes with temperature among the three GLH based texture features. (b) Average Value±Std of homogeneity in the direction of 45° changes with temperature among the sixteen GLCM based texture features. (c) Average Value±Std of correlation changes with temperature among the twelve GGCM based texture features. (d) Average Value±Std of hybrid entropy changes with temperature among the twelve GGCM based texture features.

The mean gray scale, standard deviation, and gray level entropy of tissue ultrasound B-mode images at different temperature extracted. The correlation coefficients between the parameters and temperature shown in Table 2. The correlation coefficient between the mean gray scale and temperature for 18 cases of experiments is 0.9186±0.0469, which is the largest among the three GLH-based texture features. Fig 3A shows Average-Value ± Std of the mean gray scale changes with temperature. The mean gray scale increases with temperature going up, indicating that the echo intensity rises when temperature increases.

**Table 2:** Correlation coefficients (Average-Value ± Std) between the mean gray scale, standard deviation, and gray level entropy of tissue ultrasound B-mode images with different temperature.

Texture parameters	Mean gray scale	Standard deviation	Gray level entropy
Correlation coefficients	0.9186±0.0469	-0.7924±0.1357	0.8266±0.0811

### 3.2. Correlations between GLCM parameters and temperature

The angular second moment, contrast, entropy and correlation in the ROIs of tissue ultrasound B-mode images at four directions (0°, 45°, 90°, 135°) and under different temperature were calculated. The correlation coefficients between the parameters and temperature shown in Table 3. The correlation coefficient between homogeneity in the direction of 45° and temperature for 18 cases of experiments is 0.9167 ± 0.053, which is the largest among the sixteen GLCM based texture features. As shown in Fig 3B, Average-Value±Std of homogeneity in the direction of 45° changes with temperature. There is a negative correlation between the contrast and temperature, which indicates that the contrast decreases with the increase of temperature. Both the correlation and the energy have small correlations with temperature, that is, the homogeneity has a good correlation with temperature. The homogeneity increases with temperature. As the tissue becomes more and more coagulated, the B-mode image becomes more homogeneous, which is consistent with experimental observation.

**Table 3:** Correlation coefficients (Average-Value ± Std) between gray level co-occurrence matrix (the angular second moment, contrast, entropy and correlation) at 4 directions (0°, 45°, 90°, 135°) of tissue ultrasound B-mode images with different temperature.

Angle	Contrast	Correlation	Energy	Homogeneity
0°	-0.7949±0.109	-0.2574±0.213	0.6459±0.233	0.8377±0.089
45°	-0.6499±0.152	0.4867±0.231	0.7698±0.101	0.9167±0.053
90°	-0.5894±0.127	0.6867±0.138	0.7587±0.163	0.8514±0.103
135°	-0.7642±0.193	0.5851±0.158	0.6987±0.212	0.8397±0.075

### 3.3. Correlations between GGCM parameters and temperature

The correlation coefficients between the GGCM parameters and temperature shown in Table 4 of which the three largest ones for 18 cases of experiments include the correlation, the hybrid entropy, and the inverse difference moment. They are 0.883±0.039, 0.931±0.054, and -0.879±0.091, respectively, which are the largest among the twelve GGCM-based texture features. Fig 3C and 3D show Average-Value±Std of correlation and hybrid entropy changes with temperature respectively, both of them increase with temperature.

**Table 4:** Correlation coefficients (Average-Value ± Std) between 12 texture parameters of gray level-gradient co-occurrence matrix of tissue ultrasound B-mode images with different temperature.

Parameters	Small gradient	Large gradient	Gray level nonuniformity	Gradient nonuniformity
Coefficients	0.034±0.023	0.152±0.223	-0.841±0.095	-0.312±0.161
Parameters	Gradient mean square deviation	Correlation	Gradient entropy	Hybrid entropy
Coefficients	-0.624±0.2031	0.883±0.039	0.339±0.179	0.931±0.054
Parameters	Energy	Mean gradient	Inertia	Inverse difference
Coefficients	-0.797±0.112	0.171±0.105	0.610±0.203	-0.879±0.091

Correlation coefficients of five large texture feature parameters with temperature and the mean P-value shown in Table 5. For all of these parameters, the mean and variance of this parameter at each temperature calculated for 18 experimental results. The means used to fit curves and the linear regression with temperature conducted by plotting the means and correlation curves of these parameters. The statistical results mainly presented in the form of pictures.

**Table 5:** Correlation coefficients of five large texture feature parameters of ultrasound images with temperature and their mean P-value.

Parameters	Mean gray scale	Homogeneity at 45°	Correlation
Coefficients	0.9186±0.0469	0.9167±0.053	0.883±0.039
Mean P-value	0.00	0.00	0.02
Parameters	Hybrid entropy	Inverse difference	
Coefficients	0.931±0.054	-0.879±0.091	
Mean P-value	0.01	0.02	

## 4. Discussion

Computerized algorithms for US imaging permit the detection of tissure interfaces or boundaries between anatomical structures and can provide temporal and spatial analysis for identifying the patterns and characteristics indicative of conceptual definitions of pathologies (elementary lesions). The US device performed the pre-processing of raw sound-wave signals captured to manipulate the image prior to displaying it. Techniques include the time-gain compensation of the US beam as a function of depth, edge enhancement applying filtering techniques, fill-in interpolation, and magnification technique to improve spatial detail. Some new methods developed to get organization physical information or diagnose disease through raw sound-wave signals [19, 20]. Development of most ultrasound equipment is for better imaging not for temperature estimation, this paper avoids the loss of information caused by some imaging algorithms through RF data imaging, and the results obtained are more general. Recently, there are many research reports about noninvasive temperature estimation in hyperthermia by B-mode ultrasound image processing. For example, Zhang et al. [21] showed that the small gradient preponderance, hybrid entropy and mean gray scale had a good linear correlation with temperature. Alvarenga et al [22] studied the correlation between the entropy and the correlation of the grey-level co-occurrence matrix within the temperature from 27–44°C. Yang et al [23] found that some of the texture parameters correlated with the temperature acquired from microwave ablation. Despite these benign results, the measure on B-mode images is highly sensitive to medium movements (even small) that are common in in vivo applications. In this paper, the temperature distribution of the samples was uniform due to the utilization of water bath system, it is robust and for the regions considered in our study it can maintain similar results.

The main US disadvantage lies in its considerable dependence on the sonographer’s experience and the quality of the US device. At present, ultrasound computer-aided diagnosis systems developed for multiple clinical applications. With an initial emphasis on cancer [24], ultrasound computer-aided diagnosis systems have extended into the diagnosis areas of interest [25] such as segmentation of anatomical structures and lesions [26, 27]. This work systematically examined 31 texture features to explore the most significant ones that could use for noninvasive monitoring of both MH and TA. As the results of the experiment, 5 parameters were demonstrated, including mean gray scale of GLH, entropy of GLCM, hybrid entropy, inverse difference moment, and correlation of GGCM. A linear relation observed when the tissue heated inside the water bath. These texture features analyzed for further detection of solidification zone by using classification methods such as support vector machine and neural network [27, 28].

The temperature range may not be large enough to simulate the condition of tissue ablation. When we use radiofrequency ablation to induce the coagulative necrosis, the temperature in the tissue can reach almost 100°C. The current experimental design is therefore not applicable to simulate the process of tissue ablation. The range of temperature used in this experiment includes both normal and coagulation of tissue (60°C). Despite the strong correlations between some parameters and temperature within the range from 20–54°C, their variation with temperature from 54–60°C became smaller, as shown in Fig 3B and 3D, respectively, this could be possibly due to the fact that the in vitro porcine liver tissue coagulated at 54°C. The coagulation caused the texture to change more slowly with temperature. Therefore, the parameters that have strong correlations with temperature can be used as the characteristic parameters for tissue characterization of damaged region after hyperthermia, which might offer a potential pragmatic prospect for the practice of evaluating postoperative effect of hyperthermia [29, 30].

One of the limitations of this study is that water bath cannot be used as the heating source in clinical treatment. In that case, the feasibility of the image parameters found in the current experimental configurations has to be further investigate and validated in clinical treatment. Another limitation is that the current ultrasonic signal processing and analysis was implemented offline. Therefore, the online processing should be realize to achieve noninvasive real-time monitoring of hyperthermia. The changes in some feature parameters have found to reflect the changes in tissue temperature during the heating procedure in this paper. In future works, a mathematical model based on this relationship can be established and amended by considering the influencing factors in practice. Temperature could acquire by using the model once the feature parameters calculated from the B-mode ultrasound image.

## 5. Conclusion

In summary, a novel strategy to analyze the correlations between B-mode image texture features and tissue temperature in hyperthermia developed for noninvasive monitoring of MH or TA. According to the experiments with porcine liver heated by water bath, the texture features parameters (mean gray scale of GLH, homogeneity of GLCM, hybrid entropy, inverse difference moment, and correlation of GGCM) extracted from tissue RF signal results displayed dramatic relationship to temperature. Especially, some of these parameters achieved higher than 0.9 correlation coefficients when the tissue temperature changes from 20°C to 60°C. Overall, the obtained experimental results provided solid foundation for the correlation analysis of B-mode image texture features and tissue temperature, and therefore exhibited great potentials for developing effective noninvasive monitoring during clinical hyperthermia treatments.

## Supporting information

S1 Data (RAR)
